# Supplementary figures and images for: Fusion of Normoxic- and Hypoxic-Preconditioned Myoblasts Leads to Increased Hypertrophy
Source: Cells. 2022 Mar 21;11(6):1059. doi: 10.3390/cells11061059 (PMC8947054; doi:10.3390/cells11061059)

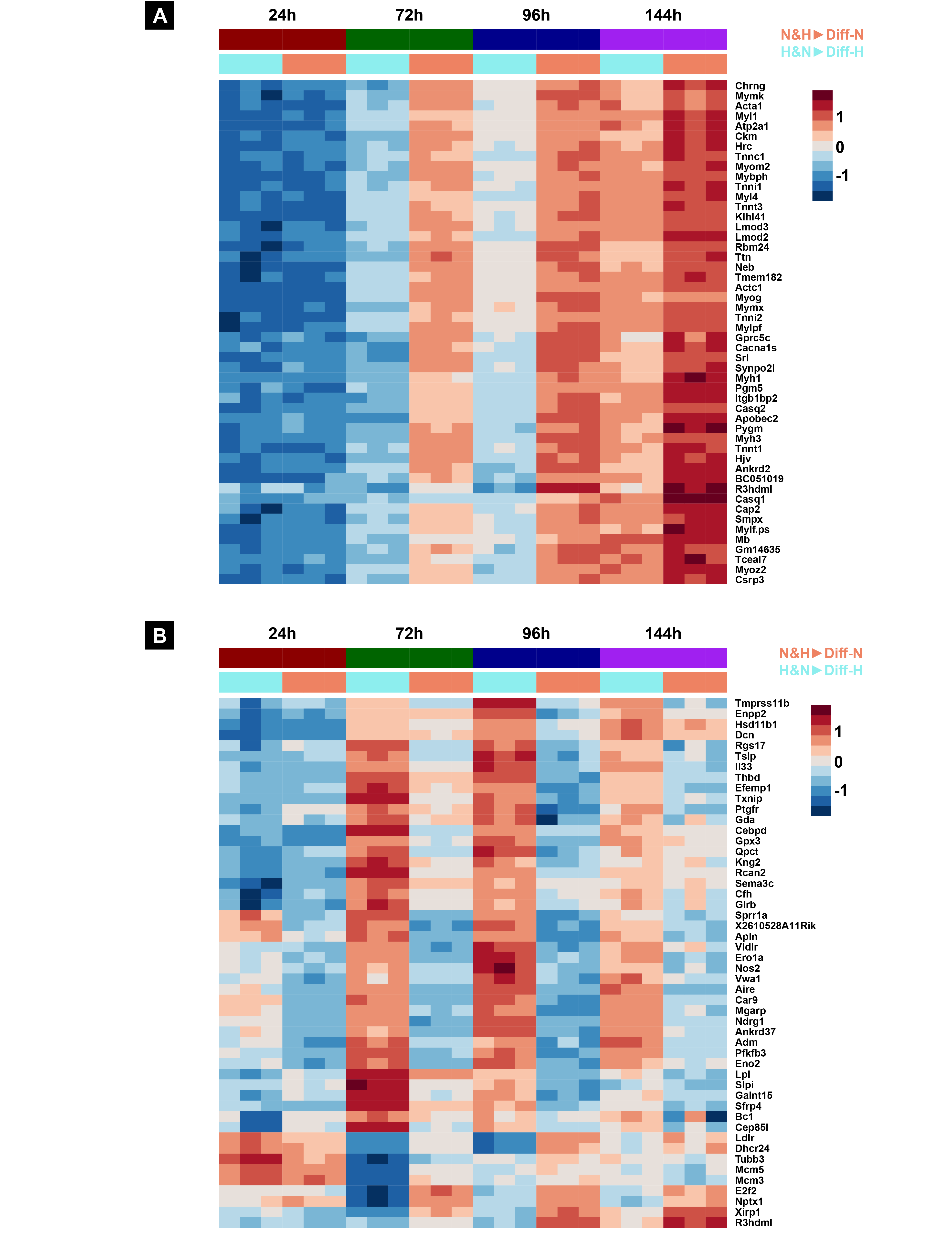

Supplement: Supplementary file 1 [file cells-11-01059-s001.zip › Pircher et al._Fig.S1.tif]

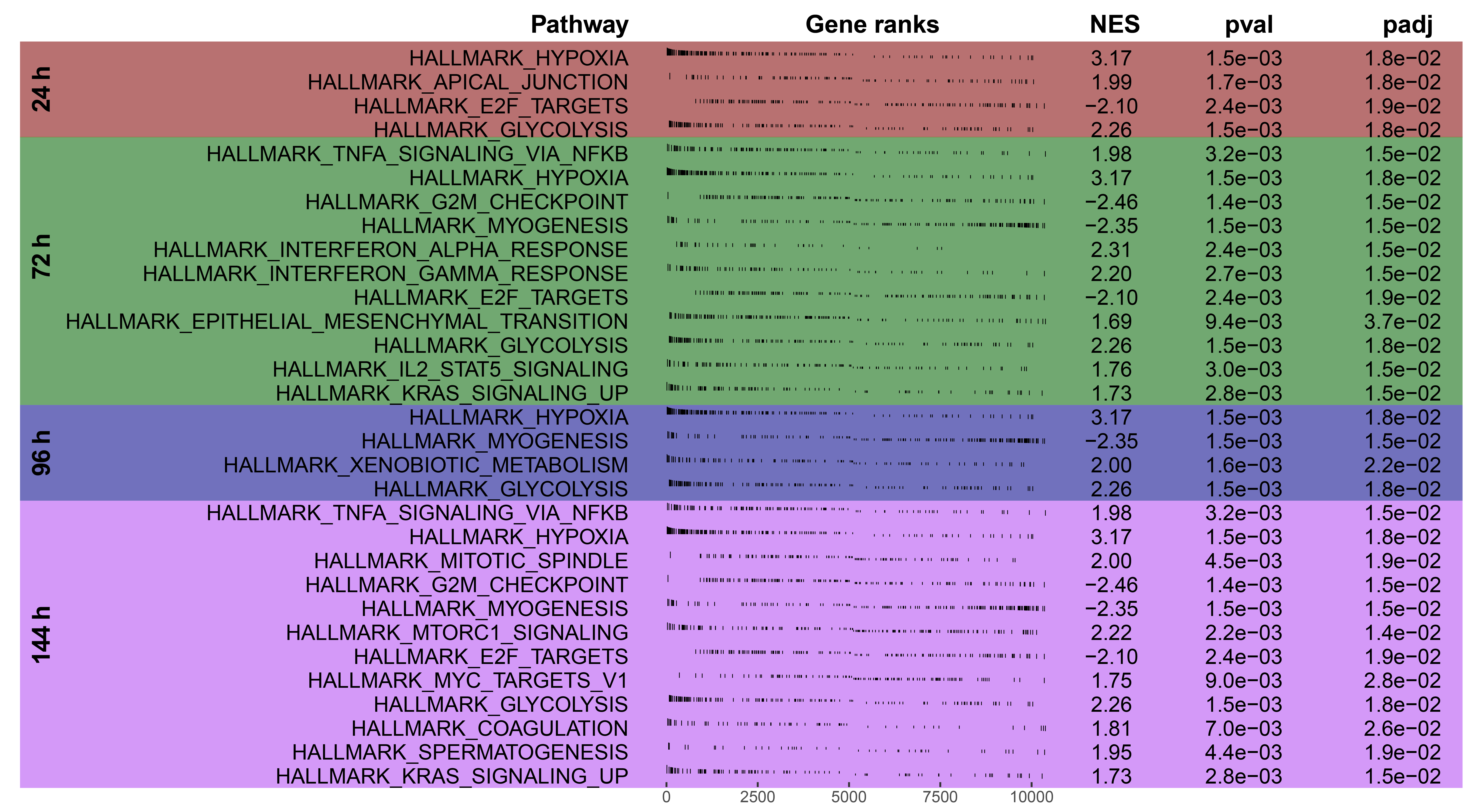

Supplement: Supplementary file 1 [file cells-11-01059-s001.zip › Pircher et al._Fig.S2.tif]
